# Limb biomechanics in combat sports: insights from wearable sensor technology

**DOI:** 10.3389/fbioe.2025.1663592

**Published:** 2025-12-05

**Authors:** Hongyu Xue, Chaoran Han, Dinghuang Zhu

**Affiliations:** 1 School of Physical Education, Shanghai University of Sport, Shanghai, China; 2 Faculty of Health Sciences and Sports, Macao Polytechnic University, Macao, China; 3 School of Athletic Performance, Shanghai University of Sport, Shanghai, China

**Keywords:** wearable sensors, biomechanics, combat sports, performance analysis, injury prevention

## Abstract

The application of wearable sensor technology in combat sports has created unprecedented opportunities for the objective, in-field analysis of limb biomechanics. This review synthesizes the current state of academic research on the use of wearable sensors for analyzing combat sports. We provide a comprehensive overview of the research landscape, identifying key sub-domains, including performance analysis, injury risk assessment, and training load monitoring. The primary sensor technologies employed are inertial measurement units (IMUs), surface electromyography (sEMG), and pressure sensors, which are increasingly used to quantify kinematic and kinetic variables of punches, kicks, and other combat-specific movements. The review details representative research findings within each sub-domain, highlighting how wearable sensors have been used to differentiate skill levels, classify techniques, estimate impact forces, and monitor fatigue. We critically examine the main challenges and controversies in the field, including the crucial issues of sensor validation against gold-standard laboratory equipment, the lack of standardized testing protocols, and the practical challenges of translating complex data into actionable insights for coaches and athletes. Finally, we offer a forward-looking perspective on the future of this interdisciplinary field, emphasizing the potential of multi-sensor fusion, advanced machine learning algorithms, and the development of smart textiles to further enhance our understanding of combat sports biomechanics. This review aims to provide a structured overview for researchers, practitioners, and technologists, while also outlining a roadmap for future investigations to overcome existing limitations and unlock the potential of wearable technology in combat sports.

## 1 Introduction

### 1.1 The convergence of combat sports science and wearable technology

The science and practice of combat sports are undergoing a profound transformation, driven by the convergence of biomechanics and wearable sensor technology (Blanco Ortega et al., 2022). Historically, the analysis of athletic performance in disciplines such as boxing, karate, Taekwondo, and mixed martial arts (MMA) has been dominated by traditional, often subjective, coaching methodologies (Saponara, 2017). The “coach’s eye,” while invaluable, is inherently qualitative and limited in its ability to discern the subtle, high-velocity biomechanical variables that determine the difference between a successful technique and a failed one. Combat sports present a unique set of measurement challenges due to their high-impact, dynamic, and unpredictable nature, which involves complex, multi-planar movements executed at maximal speeds (Ross, 2022). This environment makes traditional laboratory-based biomechanical assessment, long considered the gold standard, problematic (Nitschke, 2025; Afzal et al., 2025). This lack of ecological validity fails to capture performance under realistic conditions of physical fatigue, opponent interaction, and psychological stress, which are integral components of combat (Krabben et al., 2019).

**FIGURE 1:**
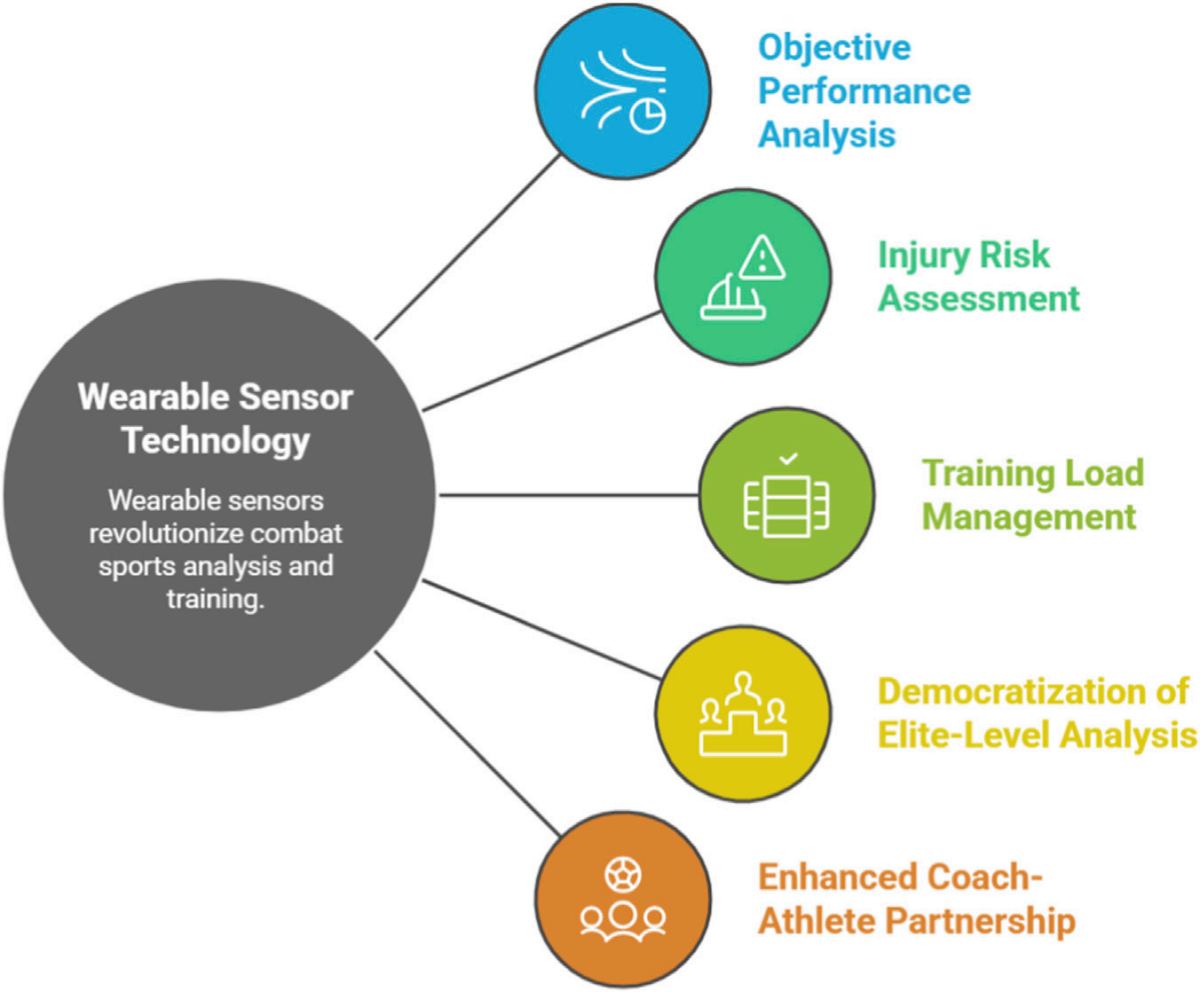
Transforming combat sports with wearable technology.

The proliferation of wearable sensor technology represents more than a mere technological upgrade; it signifies a fundamental paradigm shift in training philosophy (Ometov et al., 2021; Pearl et al., 2025). Figure 1 illustrated several potential directions for transforming combat sports with wearable technology. This evolution marks a transition from a “master-apprentice” model, reliant on qualitative observation and experience, to a more collaborative “scientist-athlete” model grounded in objective, quantifiable data. Wearable sensors are the key enablers of this shift, facilitating the migration of biomechanical analysis from the constrained laboratory to the ecologically valid environments of the training camp, sparring ring, and competition tatami (Apte, 2022). By providing objective metrics such as punch force in Newtons or limb velocity in meters per second, these technologies remove subjectivity and empower athletes and coaches to make evidence-based decisions regarding technique modification, training load management, and recovery strategies. This democratization of elite-level analysis, previously accessible only in specialized labs, is reshaping the coach-athlete dynamic into a data-informed partnership. The research landscape in this domain is propelled by a powerful, dual-pronged demand: the relentless pursuit of a competitive edge through performance enhancement and the growing concern for athlete health and career longevity, particularly regarding injury mitigation (Liu, 2024; Mosewich et al., 2014). These two drivers are not mutually exclusive but are deeply intertwined, reflecting the core priorities of modern professional sports. Improving technique to increase strike force, for instance, can concurrently lead to more efficient movement patterns that reduce chronic stress on joints, thus enhancing both performance and durability.

### 1.2 From laboratory to the field: the role of inertial measurement units (IMUs) and other wearables

The technological foundation of this revolution is the microelectromechanical system (MEMS), a chip-level innovation that has enabled the manufacturing of small, lightweight, unobtrusive, and increasingly cost-effective sensors (Bao and Wang, 1996; Chircov and Grumezescu, 2022). Among these, the Inertial Measurement Unit (IMU) has emerged as the most prevalent and versatile tool for field-based kinematic analysis in combat sports (Marković, 2022; da Costa, 2020). A typical IMU integrates a triaxial accelerometer, a triaxial gyroscope, and often a triaxial magnetometer (Faisal et al., 2019). The accelerometer measures linear acceleration, the gyroscope measures angular velocity, and the magnetometer provides orientation relative to the Earth’s magnetic field, which helps correct for gyroscopic drift (Chow et al., 2018). Together, these components allow for the calculation of a segment’s orientation, velocity, and position in three-dimensional space.

Beyond IMUs, a suite of other wearable sensors contributes to a more holistic biomechanical and physiological understanding of the combat athlete. Surface electromyography (sEMG) sensors, which measure the electrical activity of muscles from the skin’s surface, provide critical insights into muscle activation timing, intensity, and fatigue (Alcan and Zinnuroğlu, 2023; Rampichini et al., 2020). Piezoresistive and piezoelectric force and pressure sensors, which can be embedded in gloves, striking pads, or insoles, allow for the direct measurement of kinetic variables like impact force and center of pressure (Peng et al., 2024; Xu et al., 2023). Furthermore, the emerging field of smart textiles, which involves weaving conductive fibers and electronic components directly into garments, promises a future of seamless, full-body monitoring without the encumbrance of discrete sensor pods (Stoppa and Chiolerio, 2014; Mokhtari et al., 2020; Fernández-Caramés and Fraga-Lamas, 2018). A central theme throughout this review is the relationship between these field-based wearable technologies and the laboratory-based “gold standards” they seek to complement or, in some cases, replace. The validation of wearable sensor data against systems like Vicon optical motion capture and Kistler force plates is a critical and ongoing area of research that underpins the scientific legitimacy of this entire field.

### 1.3 Overview of key biomechanical parameters

To systematically analyze the contributions of wearable sensors, it is essential to define the key biomechanical parameters that are the focus of measurement. These parameters can be broadly categorized into kinetics (the study of forces causing motion) and kinematics (the description of motion itself).

#### 1.3.1 Kinetics

- Impact Force: The peak force generated during a strike, typically measured in Newtons (N). It is a primary determinant of a strike’s potential to cause damage and is a key performance indicator. Studies have shown that combat athletes can generate great forces in certain kicks (Corcoran et al., 2024).
- Impulse: Defined as force integrated over the time of impact (J = ∫Fdt), impulse reflects the total momentum change delivered to a target. A higher impulse can be more indicative of effective force transfer than peak force alone.
- Power: The rate at which work is done (P=F⋅v), representing the combination of force and velocity. It is a critical measure of explosiveness in combat sports.
- Effective Mass: A more advanced biomechanical concept representing the portion of the athlete’s body mass that is effectively transferred into the impact (Kacprzak et al., 2025). It is calculated as the ratio of peak impact force to the acceleration of the striking limb at the moment of contact and provides insight into the efficiency of the entire kinetic chain.

#### 1.3.2 Kinematics

- Linear and Angular Velocity: The speed and direction of a limb segment (e.g., hand or foot) and the rate of rotation of a joint, respectively. Maximum velocity is a crucial factor in bypassing an opponent’s defense and contributing to impact force.
- Acceleration: The rate of change of velocity. Peak acceleration of the striking limb is a key component in generating high impact forces.
- Joint Angles and Range of Motion (ROM): The measurement of joint flexion, extension, and rotation during a technique. Proper joint angles and ROM are fundamental to efficient technique, power generation, and injury prevention.
- Movement Trajectory: The three-dimensional path of a limb during a strike. Analyzing the trajectory can reveal inefficiencies or deviations from an optimal movement pattern.

**FIGURE 2:**
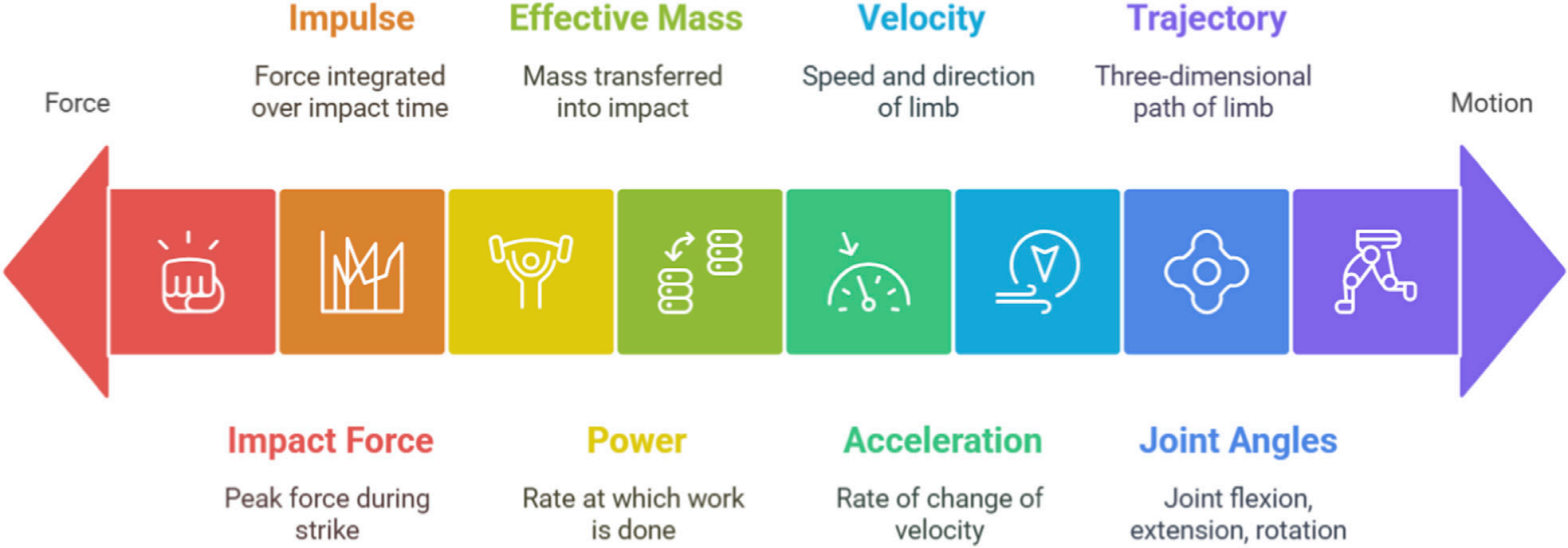
Biomechanical parameters range from force to motion description.

Understanding these parameters is fundamental, as their quantification allows for the objective evaluation of technique, the optimization of performance, and the identification of movement patterns that may increase the risk of both acute and chronic injury (Figure 2).

### 1.4 Purpose and structure of the present review

The purpose of this review is to systematically synthesize and critically analyze the current body of literature on the application of wearable sensor technology for the analysis of limb biomechanics in combat sports. By examining research across a diverse range of disciplines—including boxing, karate, Taekwondo, MMA, Judo, and Kendo—this paper aims to provide a comprehensive overview of the state of the art. The following sections will first delineate the major research foci that have emerged in this interdisciplinary field. Subsequently, a detailed synthesis of key findings will be presented, organized thematically and by sporting discipline, to highlight both common principles and sport-specific nuances. This will be followed by a critical discussion of the most significant challenges, limitations, and controversies currently facing the field, including issues of validation, standardization, and data interpretation. The review conclude with an outlook on future perspectives, exploring emerging technologies and research directions that are poised to shape the next-generation of combat sports science. This structured approach provides a potential roadmap for researchers, practitioners, and students to navigate this rapidly evolving and impactful field.

## 2 Major research foci in wearable biomechanics for combat sports

**FIGURE 3:**
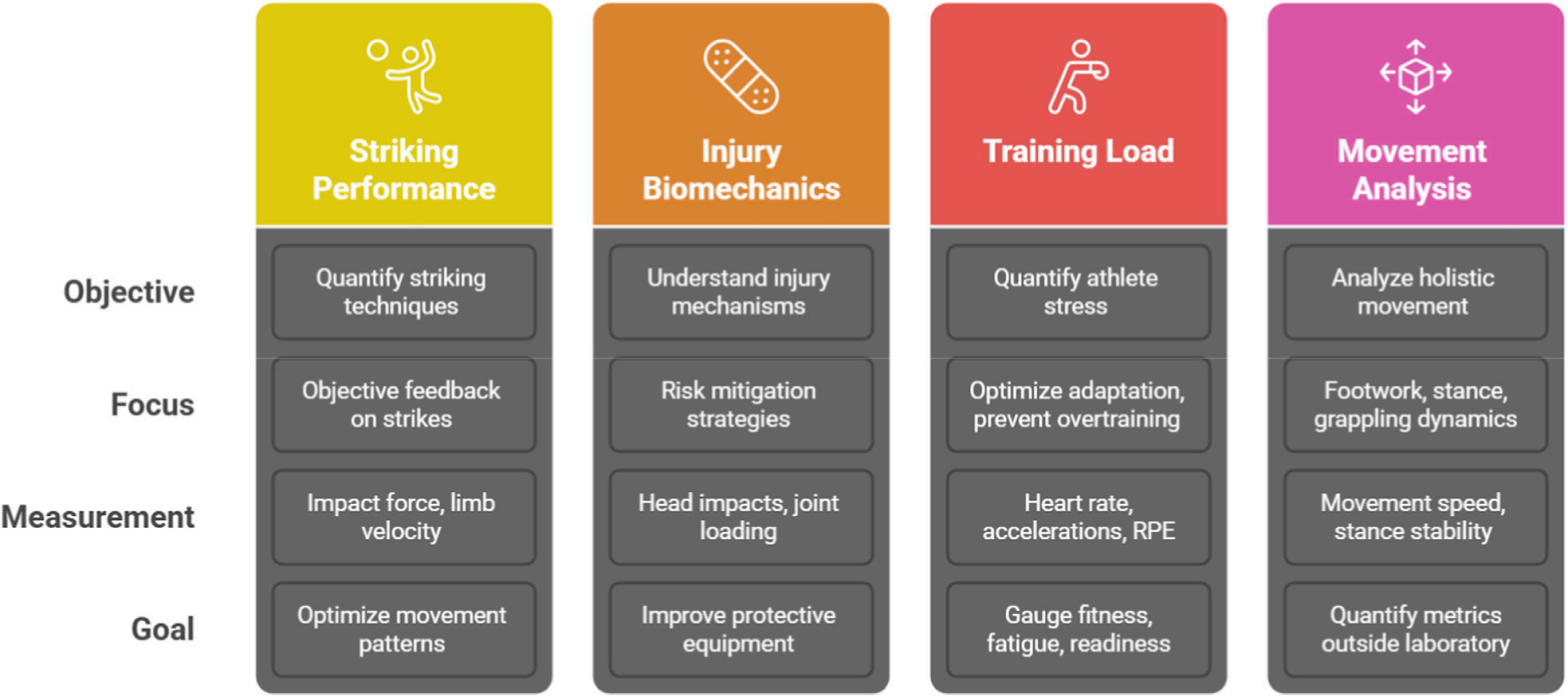
Research foci in wearable biomechanics for combat sports.

The application of wearable sensors in combat sports has coalesced into four primary research domains, each addressing a distinct aspect of athletic performance, health, and training optimization. These sub-fields, while often overlapping, provide a useful framework for categorizing the current body of scientific inquiry (Figure 3).

### 2.1 Striking performance and technique quantification

This is the most established and heavily researched domain within the field.^3^ The primary objective is to move beyond subjective coaching assessments and provide objective, quantitative feedback on striking techniques. Research in this area uses wearables to measure the *quality* and enable the *classification* of punches and kicks (Walters et al., 2025). Key performance indicators (KPIs) such as impact force, impulse, power, limb velocity, and acceleration are quantified to create biomechanical profiles of different strikes (Lenetsky et al., 2022; Muggenthaler et al., 2018). This data allows coaches and athletes to identify technical flaws, track improvements over time, and optimize movement patterns for maximum efficiency and effectiveness. A significant component of this sub-field is the use of machine learning algorithms to automatically classify different strike types (e.g., distinguishing a jab from a hook or a front kick from a roundhouse kick) based on the unique signatures present in the sensor data, thereby automating a laborious aspect of performance analysis (García et al., 2009; Cust et al., 2019).

### 2.2 Injury biomechanics and risk mitigation

This sub-field leverages wearable technology to understand the mechanisms of injury and develop strategies for risk mitigation (Rebelo et al., 2023). The research is bifurcated into two main areas of concern, reflecting the most common and severe injuries in combat sports.

The first, and more prominent, area is the study of Traumatic Brain Injury (TBI). This research employs instrumented devices such as mouthguards, headgear sensors, and skin patches to quantify the frequency, magnitude, location, and direction of head impacts sustained during sparring and competition (Wu, 2017; Schmid et al., 2021). The goal is to build a comprehensive database of head impact exposure to better understand the biomechanics of concussion, identify high-risk scenarios, and inform the development of improved protective equipment and safety regulations.

The second area focuses on Musculoskeletal Injury (MSI). This research analyzes limb and trunk biomechanics to identify risk factors for both acute and chronic injuries, such as ligament tears, tendinopathies, and stress fractures (Lee, 2016; Whiting and Zernicke, 2008). By using IMUs and pressure insoles to monitor parameters like gait, foot strike patterns, landing mechanics from jumps or throws, and joint loading during repetitive movements, researchers aim to detect aberrant or inefficient movement patterns that may predispose an athlete to overuse injuries (Meinert, 2016). This domain is closely linked to training load monitoring, as injury risk is often a function of both movement quality and accumulated physical stress.

### 2.3 Training load and fatigue monitoring

This research focus uses wearables to quantify the total stress—both physiological and biomechanical—placed on an athlete during training and competition (Halson, 2014; Adão Martins et al., 2021). The objective is to manage this stress to optimize adaptation, prevent overtraining, and ensure the athlete reaches peak condition at the appropriate time (Halson, 2014). This is typically conceptualized through the monitoring of two distinct types of load.

Internal load refers to the physiological and psychological response of the athlete to the training stimulus and is commonly measured using heart rate (HR) monitors, heart rate variability (HRV) trackers (e.g., WHOOP, Oura Ring), and subjective measures like Ratings of Perceived Exertion (RPE) (Impellizzeri et al., 2019; Ide et al., 2023).

External load refers to the physical work performed by the athlete and is quantified using biomechanical data from IMUs, such as the total number of strikes, cumulative accelerations (often expressed as “Player Load”), and distances covered (Impellizzeri et al., 2019; Ide et al., 2023). By analyzing the relationship between external and internal load, coaches can gauge an athlete’s fitness, fatigue level, and readiness to perform.

### 2.4 Movement analysis: footwork, stance, and grappling dynamics

This emerging sub-field expands the analytical focus from isolated limb movements to more holistic and foundational aspects of combat. One area of growing interest is the analysis of footwork and stance, which are fundamental to effective offense, defense, and positioning in all combat sports (Gentili, 2019). Wearable sensors, particularly IMUs placed on the feet or lower back, are beginning to be used to quantify metrics like movement speed, stance stability, and specific footwork patterns (e.g., pivots, lateral shuffles), which have historically been difficult to measure outside of a laboratory (Lopez-Nava and Munoz-Melendez, 2016).

A second, and significantly more challenging, area is the biomechanical analysis of grappling (Blanco Ortega et al., 2022). This includes the study of throws and takedowns in sports like Judo, Wrestling, and MMA, as well as ground control and submission techniques (Dal Bello et al., 2019). This domain remains severely under-researched with wearable sensors due to the immense methodological difficulties. The close, dynamic interaction between two bodies makes it challenging to place sensors in locations where they will not be dislodged or interfere with the athletes’ movements. Sensor displacement can introduce artifacts in the data and pose a risk of discomfort or injury unrelated to the athletic activity Furthermore, defining and interpreting meaningful biomechanical metrics for complex, continuous grappling exchanges is far more difficult than for discrete striking actions.^15^ This represents a significant frontier for future research.

To provide a consolidated overview of the technologies underpinning these research foci, Table 1 summarizes the primary wearable sensor types, the biomechanical parameters they measure, and their typical applications in combat sports.

**TABLE 1:** Summary of wearable sensor technologies and their application in combat sports biomechanics.

Sensor type	Primary biomechanical parameters measured	Example combat sport application
Inertial measurement unit (IMU)	Linear/Angular acceleration, velocity, orientation, joint angles, range of motion (ROM)	Quantifying punch/kick velocity and trajectory in boxing and Taekwondo; analyzing footwork patterns; assessing throwing kinematics in Judo
Surface electromyography (sEMG)	Muscle activation timing, intensity, duration, fatigue	Analyzing the muscle firing sequence in a karate punch; assessing muscle fatigue during repeated strikes; comparing muscle contributions between elite and novice athletes
Piezoresistive/Piezoelectric sensor	Impact force, impulse, pressure, center of pressure	Measuring the impact force of a punch on a bag or glove; analyzing grip pressure distribution on a Kendo sword; quantifying ground reaction forces
Pressure insoles	Plantar pressure distribution, ground contact time, foot strike pattern	Analyzing foot pressure during the ginga movement in Capoeira; assessing balance and weight transfer during throwing techniques
Instrumented mouthguard	Head linear/Angular acceleration, impact location, impact duration	Quantifying head impact exposure in MMA and boxing sparring to study TBI risk; validating other head-worn sensors
Smart Textiles/Strain sensors	Joint angles, ROM, respiration rate, muscle deformation	Seamlessly monitoring full-body kinematics without discrete sensors; measuring chest expansion for breathing analysis during exertion

## 3 State of the art: a discipline-specific and thematic synthesis

**FIGURE 4:**
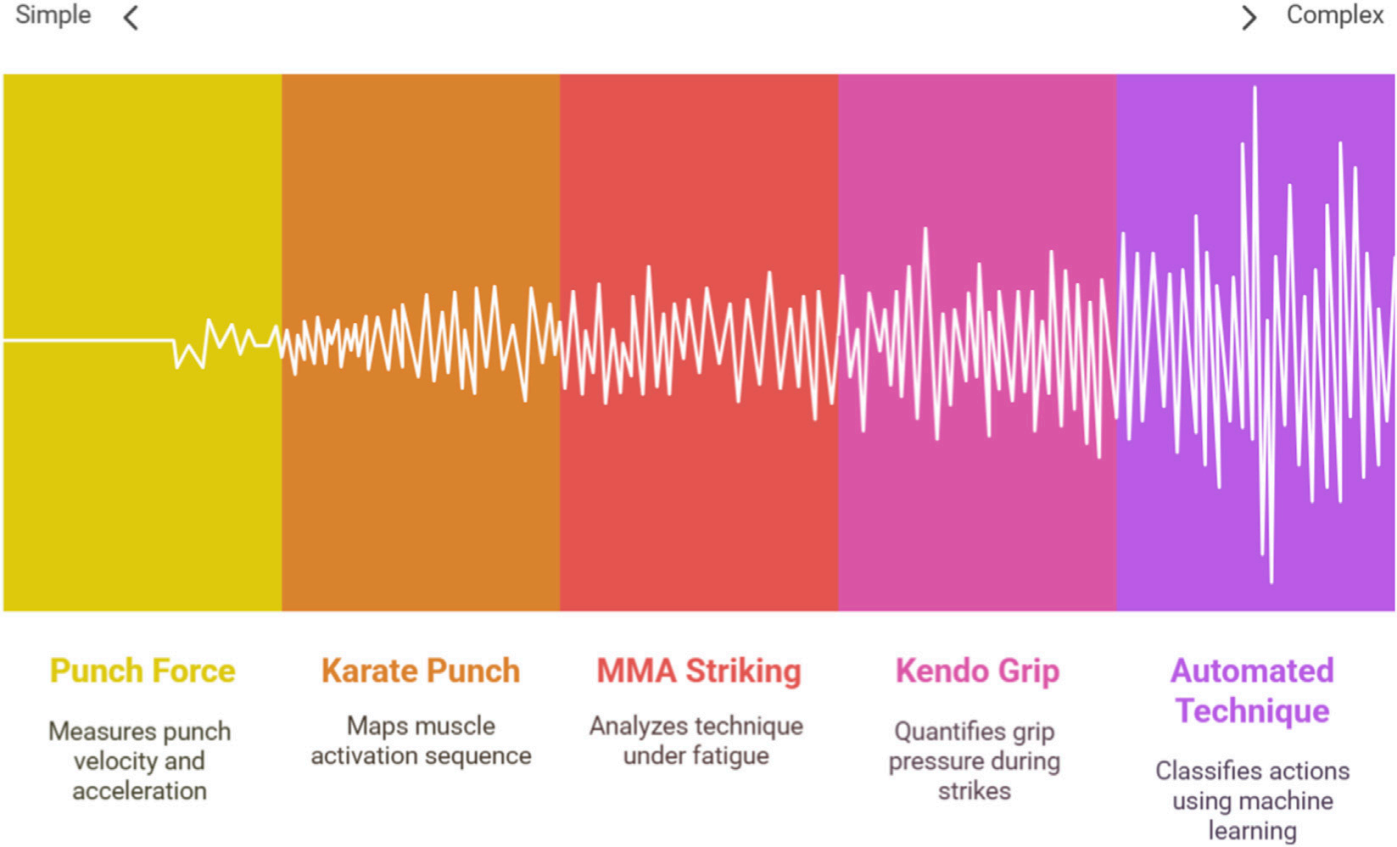
Wearable sensor research ranges from simple to complex analysis.

This section synthesizes the specific findings reported in the literature, organized according to the major research foci and contextualized within specific combat disciplines. This approach illuminates both the universal biomechanical principles and the sport-specific nuances revealed by wearable sensor technology. Figure 4 shows the different scenarios of research on wearable sensors from simple to complex analyses.

### 3.1 The biomechanics of striking: insights from boxing, karate, and Taekwondo

Striking-dominant sports have been the most fertile ground for wearable sensor research, primarily because strikes are discrete, ballistic events that are relatively straightforward to instrument and analyze compared to the continuous, multi-body interactions of grappling (Gavagan and Sayers, 2017).

#### 3.1.1 Upper limb dynamics: Punch force, velocity, and the kinetic chain

The quantification of punching performance has progressed from rudimentary dynamometers to sophisticated, validated wearable systems (Haralabidis et al., 2020). Modern research extensively uses IMUs, often integrated directly into gloves or worn on the wrist, to measure kinematic variables like punch velocity, acceleration, and trajectory (Connolly et al., 2017). Validation studies comparing these wearable systems against gold-standard laboratory equipment, such as Kistler force plates and Vicon optical motion capture, have demonstrated remarkably high accuracy (Harrison et al., 2024). This high fidelity allows researchers and practitioners to confidently use these tools in the field to establish performance benchmarks, with studies identifying typical velocity ranges for different punches (e.g., jab, cross, hook) across various skill levels (Haralabidis et al., 2020; Worsey et al., 2020; Ye et al., 2022).

More advanced research has moved beyond simply measuring the motion of the fist to investigating how the entire body contributes to the impact—a concept embodied by the kinetic chain (Chu et al., 2016). A pivotal parameter in this context is “effective mass,” defined as the ratio of peak impact force to the fist’s acceleration at contact. It quantifies how efficiently an athlete integrates their body mass into the strike. A recent, detailed study on trained boxers revealed a crucial insight: straight punches (jab and cross) exhibit significantly higher effective mass than hook punches (Kacprzak et al., 2025). This indicates that straight punches, which rely on a more linear force transmission pathway, are more biomechanically efficient at transferring mass into the target, even though hooks may sometimes generate higher peak forces. This finding underscores that superior technique, which optimizes the coordination of the kinetic chain, is more critical for effective force transfer than an athlete’s absolute body mass or muscularity (Kacprzak et al., 2025).

Complementing kinetic and kinematic analyses, surface electromyography (sEMG) provides a window into the neuromuscular control underpinning these movements (Scano et al., 2024). Studies on the karate punch (*choku-zuki*) have used sEMG to map the precise sequence of muscle activation. This research confirms a proximal-to-distal firing pattern, where the larger muscles of the shoulder and torso (e.g., pectoralis major, deltoid) activate before the muscles of the forearm (VencesBrito et al., 2011). Critically, comparisons between experienced karateka and novices reveal significant differences in neuromuscular strategy; experts exhibit more refined activation timings and shorter muscle contraction periods, indicative of greater neuromuscular efficiency and coordination honed through years of practice (Grangeiro, 2019).

#### 3.1.2 Lower limb dynamics: Kick velocity, impact force, and segmental contributions

Kicking strikes, the cornerstone of sports like Taekwondo, Karate, and Muay Thai, generate some of the most powerful forces in human movement (Gavagan and Sayers, 2017). A comprehensive systematic review reported that kicking foot velocities can range from 5.2 m/s to a staggering 18.3 m/s, with mean impact forces documented from 122.6 N to over 9015 N—a force sufficient to cause bone fractures (Corcoran et al., 2024; Marques Junior, 2020).^31^ Among the various techniques, the roundhouse kick (*dollyo chagi* in Taekwondo, *mawashi geri* in Karate) typically exhibits the highest peak velocity, while the linear side kick (*yeop chagi* or *yoko geri*) often produces the greatest impact force, highlighting a trade-off between speed and power depending on the technique’s mechanics (Diniz et al., 2021).

Wearable sensors have been instrumental in dissecting the intricate biomechanics of these powerful movements. In Taekwondo, for example, analysis of the “sine-wave” motion has revealed how practitioners utilize the lowering and raising of their center of mass to convert gravitational potential energy into kinetic energy, thereby optimizing the final impact force of a punch (Qureshi, 2020; Ma et al., 2021). Comparative studies between elite and sub-elite Taekwondo athletes consistently show that the elite group produces faster kick execution times, greater linear and angular velocities of the leg segments, and higher ground reaction forces, providing objective markers of expertise (Chen et al., 2015).

A critical methodological consideration highlighted by wearable sensor research is the influence of the target. A study on Kyokushin karate practitioners using a combination of IMUs and sEMG found that the presence of a target fundamentally altered the movement’s kinematics (Mosler et al., 2025). Striking a physical pad nearly doubled the peak acceleration of the foot segment compared to performing the same kick in the air. Interestingly, the underlying muscle activation patterns (EMG) remained largely consistent, suggesting that the central motor program was similar, but its execution was modulated by the anticipation of impact. This finding has profound implications for research design, suggesting that studies conducted in a “shadow boxing” or non-contact context may not accurately reflect the biomechanics of a true impact strike.

The application of wearables has also allowed for the objective quantification of stylistic differences within a single martial art (Pang et al., 2024). In karate, athletes specialize in either *kata* (pre-arranged forms) or *kumite* (sparring). Research using inertial sensors has substantiated long-held coaching wisdom by showing that *kumite* specialists exhibit greater mobility in the shoulder and hip joints, which is necessary for reactive, high-velocity striking. In contrast, *kata* specialists display superior ankle mobility and better static balance, particularly with their eyes closed, reflecting the discipline’s emphasis on deep stances, precise control, and proprioceptive awareness (Molinaro et al., 2020).

### 3.2 The complex demands of mixed martial arts (MMA)

MMA presents a uniquely challenging yet “ecologically valid” environment for biomechanical analysis because it involves a chaotic and unpredictable interplay of striking, grappling, and transitions between the two, all performed under extreme fatigue (Skalski et al., 2025).

#### 3.2.1 Striking under duress: Influence of fatigue and balance disruption

The ability to maintain technique and power output under fatigue is a hallmark of elite MMA fighters. A pivotal study investigated this by comparing striking biomechanics in elite fighters under three conditions: normal, fatigue-induced, and with vestibular (balance) disruption (Skalski et al., 2025). Using both 2D video and 3D motion capture, the study found that fatigue had a significant detrimental effect, decreasing both punch velocity and impact force while markedly increasing the variability of joint angles at the shoulder and elbow. This increased variability is a clear indicator of degraded neuromuscular control. However, a key finding for field-based practitioners was that even under these disruptive conditions, 2D video analysis provided reliable and valid measurements of sagittal-plane joint angles, showing excellent agreement with the 3D gold standard (Intraclass Correlation Coefficient, ICC = 0.81–0.99). This validates the use of accessible tools like smartphone cameras for practical, in-the-field assessment of striking mechanics by coaches (Skalski et al., 2025).

#### 3.2.2 The under-researched domain: Biomechanics of grappling and transitions

While striking biomechanics in MMA is a growing area of research, the analysis of grappling with wearable sensors remains a significant gap in the literature (Blanco Ortega et al., 2022). The fluid transitions from striking to takedowns and ground control, which are the signature of dominant MMA grapplers like Khabib Nurmagomedov and Cain Velasquez, are exceptionally difficult to quantify. The primary challenges are methodological: placing sensors in a way that can withstand the intense friction and pressure of grappling exchanges without being dislodged or hindering movement, and developing algorithms capable of interpreting the complex, continuous, multi-body data to extract meaningful biomechanical parameters (Worsey et al., 2019). Preliminary work using laboratory-based motion capture and force sensors to model a range of martial arts movements, including grappling, has suggested that grappling techniques are characterized by high muscle force and movement efficiency but relatively low impact forces, highlighting their emphasis on control over ballistic impact (Suo et al., 2024; Hons and Tiernan, 2020). This domain represents a major frontier for wearable sensor innovation.

### 3.3 Quantifying performance in grappling and weapon-based arts

While grappling research is challenging, some progress has been made in pure grappling arts like Judo, and innovative applications have emerged in weapon-based arts like Kendo.

#### 3.3.1 Judo: Throwing mechanics and training load

The application of wearable sensors in Judo is still in its early stages but shows significant promise (Mañas-Paris et al., 2022). Researchers have used IMUs to develop and propose objective indices for quantifying the motor abilities involved in throwing techniques, creating metrics for both force expression and coordination capabilities (Frassinelli et al., 2019). In a practical application, a study combined IMUs with physiological sensors (heart rate monitors) to evaluate a training intervention in young judokas. The results objectively demonstrated that a 3-week program of high-intensity interval training (HIIT) and plyometrics led to significant improvements in performance on the Special Judo Fitness Test (SJFT) and an increase in the peak angular velocity of their throws, as measured by the IMUs (Mañas-Paris et al., 2022). Despite these advances, much of the foundational biomechanical research on specific phases of Judo throws, such as *kuzushi* (the act of unbalancing the opponent), still relies on traditional laboratory setups with force plates and optical motion capture. This highlights a clear opportunity for the development and application of robust wearable sensor systems to bring this analysis into the dojo. The current literature also reveals a scarcity of studies on adult judoka, indicating another area ripe for investigation (Mañas-Paris et al., 2022).

#### 3.3.2 Kendo: Grip pressure dynamics during strikes

A stellar example of targeted and innovative sensor application comes from the study of Kendo, the Japanese art of fencing. By instrumenting the *shinai* (bamboo sword) with custom-built pressure sensors, researchers were able to investigate the nuanced dynamics of grip pressure during an attack (Jeong et al., 2023; Jeong et al., 2015). This research provided the first objective, quantitative evidence for the existence of the five distinct phases of a Kendo attack as taught in traditional pedagogy: *Kamae* (stance), *Seme* (initiation/pressure), *Toraeru* (opportunity capture), *Datotsu* (strike), and *Zanshin* (follow-through). The sensors revealed a unique and consistent pressure profile for each phase, such as a significant pressure drop during the *Toraeru* phase before the final strike (Jeong et al., 2023). Furthermore, the study scientifically validated the common coaching tenet that the left hand should be the dominant hand, generating higher pressure for most strikes. However, it also uncovered a crucial and surprising exception: for the thrusting attack to the throat (*Tsuki-bu*), the right hand generated significantly more pressure during the strike phase (Jeong et al., 2023). This work exemplifies the power of wearable technology to not only confirm traditional knowledge but also to reveal subtle, data-driven insights that can refine coaching and technique.

### 3.4 Automated technique recognition via machine learning

A major thrust in the application of wearable sensors is the automation of performance analysis, particularly technique classification. This approach uses the power of machine learning (ML) to bypass the need for laborious and time-consuming manual video analysis or data labeling (Worsey et al., 2020; Manoharan et al., 2025).

#### 3.4.1 From raw data to classified actions

The process involves capturing raw, time-series data from IMUs—typically triaxial acceleration and triaxial angular velocity—during a series of movements. This data stream contains unique signatures or “fingerprints” for each distinct technique. By training an ML model on a labeled dataset (where an expert has identified which segments of data correspond to which technique), the model learns to recognize these patterns (Chawla and Karakoulas, 2005; Ng et al., 2023). Once trained, the model can automatically classify new, unlabeled data streams, providing a real-time or post-session count and classification of every technique performed (Kaur and Kumar, 2021).

#### 3.4.2 Classification models and performance

A variety of ML models have been successfully employed for this task. In Taekwondo, Support Vector Machines (SVMs) have proven remarkably effective, with one study achieving over 96% accuracy in classifying four distinct kick types (front, roundhouse, side, back) using data from just a single accelerometer worn on the athlete’s waist (Liu et al., 2024). This demonstrates the potential for highly accurate classification with a minimally intrusive sensor setup. In boxing, both SVMs with a Gaussian radial basis function (rbf) kernel and multi-layered perceptron neural networks (MLP-NN) have achieved high accuracy of strike classification using data from two wrist-worn IMUs (Worsey et al., 2020; Manoharan et al., 2025).

As the field matures, more complex deep learning architectures are being deployed. Models such as Convolutional Neural Networks (CNNs), which are adept at learning from spatial patterns in data, and Long Short-Term Memory (LSTM) networks, which excel at interpreting time-series sequences, are showing great promise. One study that used a full-body suit of 17 IMUs to capture Taekwondo *Poomsae* (forms) movements achieved a classification accuracy of 97.8% with a CNN-based model (Jaya et al., 2022). Another study used a CNN-Bidirectional LSTM (CNN-BiLSTM) model to detect abnormal kicking techniques, achieving 95.67% accuracy (Jaya et al., 2022). The increasing sophistication of these models is enabling more nuanced and accurate automated analysis.

#### 3.4.3 Multimodal approaches

The most advanced systems move beyond a single data type and fuse information from multiple sources to improve robustness and efficiency. A novel framework, dubbed the “Smart Boxer” system, provides a compelling example (Menzel and Potthast, 2021a). This system integrates data from wrist-worn IMUs with synchronized video footage. Critically, it employs an active learning strategy known as “Query by Committee” to dramatically reduce the amount of manual data labeling required. The system identifies data points where its committee of ML models is most uncertain and prompts a human expert to label only those points. Using this method, the system achieved over 91% accuracy for punch recognition and over 92% for punch classification while requiring only 15% of the dataset to be manually labeled, a significant improvement in efficiency over traditional supervised learning. Other research lines are exploring whether feeding ML models derived biomechanical data, such as calculated body joint angles, can yield higher classification accuracy than using raw sensor data directly (Ashour et al., 2024). These multimodal and intelligent learning approaches represent the cutting edge of automated combat sports analysis.

## 4 Critical challenges and current controversies

**FIGURE 5:**
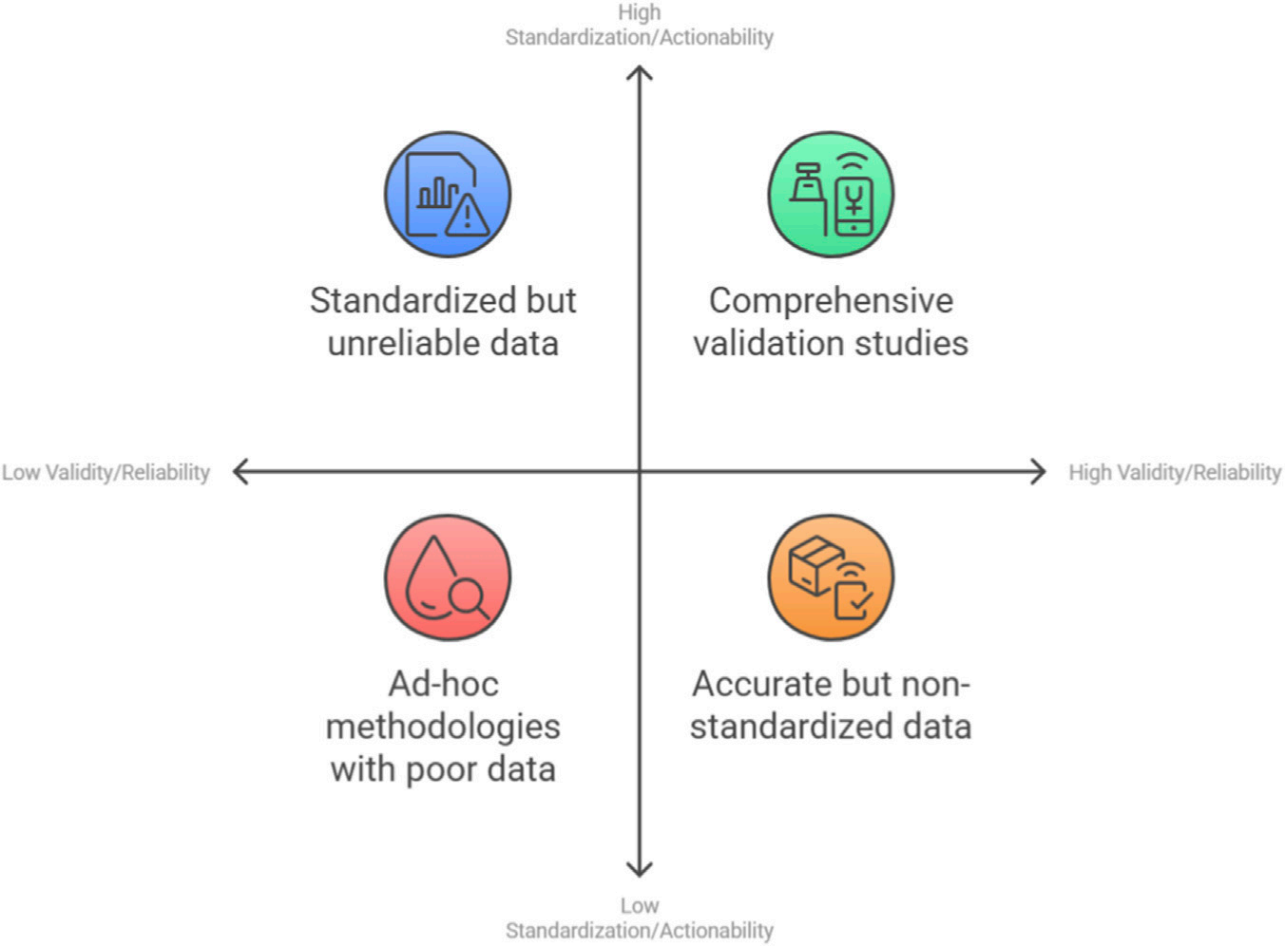
Challenges and controversies in wearable sensor application.

Despite the rapid advancements and promising results, the application of wearable sensors in combat sports biomechanics is fraught with significant challenges and ongoing debates (Figure 5). A critical evaluation of these issues is essential for guiding future research and ensuring the responsible implementation of these technologies.

### 4.1 The validity and reliability hurdle: Bridging the gap between wearables and gold standards

The foundational challenge for the entire field is the question of data integrity: are the measurements provided by wearable sensors accurate and reliable? Despite the proliferation of both commercial and research-grade devices, a substantial portion of research utilizes them without rigorous validation against established laboratory gold standards, such as 3D optical motion capture systems (e.g., Vicon) and force plates (e.g., Kistler) (Menzel and Potthast, 2021b). This practice undermines the scientific credibility of the findings and raises questions about the interchangeability of data from different systems (Pezenka and Wirth, 2025).

Numerous validation studies have been conducted, with mixed results that highlight the complexity of this issue. On the one hand, some highly specialized, custom-developed systems have shown excellent agreement with gold standards. For instance, Menzel and Potthast (2021a) conducted a comprehensive validation of their novel boxing sensor system, reporting very high correlations for punch force (R2 = 0.97–0.99) when compared directly against a Kistler force plate and Vicon motion capture setup (Menzel and Potthast, 2021a). On the other hand, many studies, particularly those using lower-cost or general-purpose devices, report more moderate or even poor reliability. A study developing a low-cost punch and kick velocity measurement system using a smartphone’s accelerometer reported only moderate reliability (ICC = 0.746–0.786) and, crucially, was not validated against a gold-standard reference, limiting its findings to reliability rather than absolute accuracy (Pezenka and Wirth, 2025).

This discrepancy in validation outcomes stems from numerous sources of error inherent to wearable technology. Soft-tissue artifact—the movement of the sensor on the skin relative to the underlying bone—is a primary concern, as it introduces noise that is not representative of true skeletal motion (Rong and Kuo, 2024).

Sensor drift, particularly the integration error that accumulates in accelerometers and gyroscopes over time, can lead to significant inaccuracies in position and orientation estimates without proper correction algorithms (e.g., sensor fusion with magnetometers) (Rong and Kuo, 2024; Kumar et al., 2013). Furthermore, the high-velocity, high-impact nature of combat sports demands very high

Sampling rates to avoid under-sampling and accurately capture the peak characteristics of an impact; low sampling rates can miss these crucial data points entirely (Hasanin et al., 2019) Finally, the process of calculating kinematic variables like velocity and displacement requires numerical integration of acceleration data, a process that notoriously amplifies any noise present in the original signal, leading to compounding errors (Pezenka and Wirth, 2025). These technical hurdles mean that validity is not a property of a sensor itself, but of the entire measurement system—sensor, attachment method, and processing algorithm—applied to a specific task.

### 4.2 Methodological standardization: the need for coherent protocols and reporting

A pervasive criticism leveled against this field is the widespread use of *ad-hoc* methodologies, which severely limits the comparability, reproducibility, and generalizability of findings (Worsey et al., 2019). There is a pressing need for the community to develop and adopt standardized guidelines for several key aspects of research design and reporting.

Many studies fail to provide adequate technical specifications of the wearable devices used, omitting critical details such as the sensor’s measurement range, resolution, sampling frequency, and the specifics of the filtering and processing algorithms applied. Furthermore, the method of sensor attachment—a crucial factor influencing data quality due to soft-tissue artifacts—is often poorly described. This lack of detailed reporting makes it nearly impossible for other researchers to replicate a study or to accurately compare findings across different investigations.

Even seemingly minor variations in experimental protocol can have a profound impact on results. For example, some studies on striking mechanics explicitly forbid the use of footwork or significant trunk rotation to standardize the movement and isolate the action of the limb (Pezenka and Wirth, 2025). While this improves intra-subject reliability, it drastically reduces the ecological validity, as elite fighters rely on the entire kinetic chain, beginning from the ground up, to generate power. Similarly, in head impact research, the choice of the g-force threshold for recording an event (e.g., a 10 g vs. a 15 g trigger) can fundamentally alter the resulting dataset, with lower thresholds capturing a higher frequency of impacts but yielding a lower average magnitude (O'Connor et al., 2017). Without standardized protocols, it becomes difficult to determine whether differences observed between studies are due to true biomechanical phenomena or are merely artifacts of disparate methodologies.

### 4.3 From data to actionable insight: the challenge of data processing, interpretation, and real-time feedback

The advent of wearable sensors has solved one problem—data collection in the field—but has created another: the “data deluge.” These devices can generate enormous, high-dimensional datasets that are often noisy and complex (Anikwe et al., 2022; Williamson et al., 2015). The critical challenge is no longer just acquiring data, but transforming it into information that is meaningful, interpretable, and, most importantly, actionable for a coach or athlete who may not have a background in signal processing or data science.

This challenge is compounded by the “black box” problem associated with advanced machine learning models (Rudin, 2019). While a deep learning model may classify a punch as a “hook” with high accuracy, it often cannot explain why it made that classification in biomechanical terms (Worsey et al., 2020). This lack of explainability can be a significant barrier to adoption, as coaches need to understand the underlying technical reasons for a performance outcome to provide effective feedback.

Despite numerous challenges, several elite training institutions have successfully integrated wearable data into daily practice. For instance, a national boxing team has deployed an accelerometer-based wearable smart punching analysis system to capture metrics such as punch count, speed, force, and power. Furthermore, the device supports exchange unit statistics, providing real-time feedback on the number of engagements, duration, intervals, and punch volume between athletes during each match, thereby offering a comprehensive overview of the overall training load.

Furthermore, a key promise of wearable technology is the provision of real-time biofeedback to guide motor learning and technique correction during a training session (Umek et al., 2015). However, the engineering and human-computer interaction challenges involved are substantial. These challenges make it difficult for traditional computer architectures and interaction methods to fully leverage the potential of new sensor algorithms in combat sports analysis, necessitating further optimization of hardware architectures and interaction designs to align with technological advancements. A practical real-time system must be computationally efficient enough to process sensor data, run a predictive or classification model, and deliver feedback—whether auditory, visual, or haptic—all within milliseconds. Crucially, this feedback must be intuitive and simple enough that it enhances, rather than disrupts, the athlete’s focus and cognitive load (Umek et al., 2015; Bowman et al., 2021).

### 4.4 Head impact monitoring: the controversy of clinical utility vs. exposure tracking

Perhaps the most contentious and widely debated topic in this field is the use of wearable sensors for head impact monitoring. The central controversy revolves around their clinical utility. A strong and consistent consensus has emerged in the scientific literature: current wearable sensors cannot and should not be used to diagnose a concussion (Le Flao et al., 2024). This conclusion is based on several factors.

First, the accuracy and validity of these devices are highly variable. A comparative study of three different sensor types (instrumented mouthguard, skin patch, headgear patch) against video-verified impacts in boxing found wildly different performance profiles (Le Flao et al., 2024). While the positive predictive value (the probability that a sensor-recorded event was a true impact) was high for all devices (>96%), their sensitivity (the ability to detect a true impact) varied dramatically, from a low of 35% for the mouthguard to 86% for a skin patch. Specificity (the ability to correctly identify non-impact events) also varied. Some commercially available systems, such as the X-Patch, have been shown in independent validation studies to suffer from extremely high false-positive rates, recording many non-impact events (like jumping or falling) as head impacts, rendering their data clinically unreliable (O'Connor et al., 2017).

Second, concussion is a complex neurophysiological event, and the risk is influenced by a multitude of factors beyond the simple biomechanics of a single impact. These include an individual’s concussion history, age, sex, genetic predispositions, and even the rotational components of the impact, which are particularly difficult to measure accurately (O'Connor et al., 2017). No single kinematic measure (e.g., peak linear acceleration) has been shown to have sufficient sensitivity and specificity to serve as a reliable diagnostic threshold.

This has led to a critical shift in how the role of these sensors is understood. The scientific community has moved away from the idea of a “concussion detector” and toward the concept of an exposure monitoring tool. The accepted utility of these sensors lies in their ability to quantify an athlete’s cumulative head impact burden over a practice, a game, a season, or an entire career (Le Flao et al., 2022). This epidemiological data is invaluable for research into the long-term consequences of repetitive head trauma (including sub-concussive impacts) and for informing evidence-based rule changes and modifications to training practices, such as limiting the amount of full-contact sparring (O'Connor et al., 2017). This distinction is not merely academic; it has profound real-world safety implications. The marketing of these devices to consumers, teams, and parents often creates the perception of a diagnostic safety net. This creates a dangerous potential for misuse, where a coach might wrongly clear an athlete to return to play because a sensor did not trigger an alarm (a false negative), or unnecessarily remove a healthy athlete due to a false positive. This tension between the cautious, evidence-based position of the research community and the commercial market’s demand for a simple “concussion alarm” remains a significant challenge requiring better science communication and potentially regulatory oversight.

## 5 Future perspectives and conclusion

**FIGURE 6:**
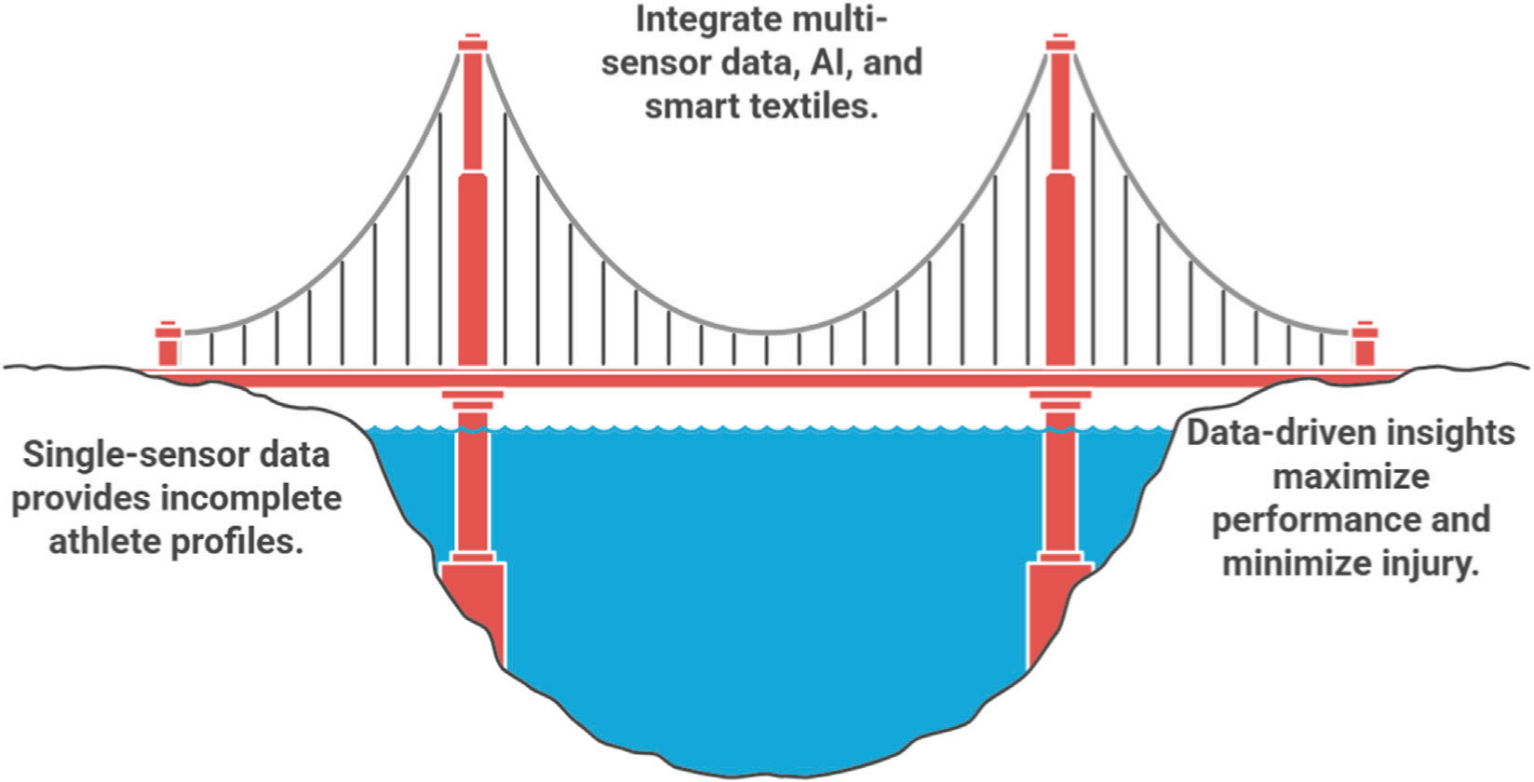
Wearable sensors evolve combat sports: From basic tracking to personalized, predictive analysis.

The field of wearable sensor technology in combat sports biomechanics is on a trajectory of rapid evolution. While significant challenges remain, emerging technological advancements and novel research paradigms are poised to overcome current limitations and unlock new frontiers of understanding in human performance and safety (Figure 6).

### 5.1 The next frontier: multi-sensor fusion and AI-driven predictive models

The future of biomechanical analysis lies not in isolated measurements from single sensors, but in the intelligent fusion of data from multiple, heterogeneous sources (Mendes et al., 2016; Bijalwan et al., 2021) and the synergistic development of the two interconnected paradigms of explainable and predictive artificial intelligence. Multi-sensor fusion is a methodology that combines data from different sensor types—for example, integrating kinematic data from IMUs, muscle activation data from sEMG, and kinetic data from pressure sensors—to construct a more complete and robust biomechanical profile of the athlete (Gravina et al., 2017; Yin et al., 2025; Gharibo, 2021). This approach holds the potential to overcome the inherent limitations of any single sensor type. For instance, fusing IMU data with biomechanical constraints and advanced algorithms can improve the accuracy of joint angle estimation and mitigate sensor drift (Xie and Zhang, 2024). This holistic approach is particularly critical for tackling the field’s most complex challenges, such as analyzing the dynamic, multi-body interactions of grappling or the fluid transitions in MMA, where a single sensor’s data is insufficient.

Concurrently, the role of artificial intelligence (AI) and machine learning (ML) is set to evolve from descriptive and classificatory functions to predictive and prescriptive analytics (K et al., 2022). Current ML models excel at classifying a strike that has already occurred (Moore et al., 2020). The next-generation of models will leverage vast longitudinal datasets to forecast future events. For example, an AI system might analyze subtle degradations in a boxer’s punching kinematics and footwork over several rounds to predict their decreasing capacity to defend, or it might identify minute changes in a fighter’s gait and landing mechanics over a training camp to generate a probabilistic forecast of their risk for a specific musculoskeletal injury (Khasanshin, 2021). This shift from reactive analysis to proactive prediction will enable coaches and medical staff to intervene before performance significantly drops or an injury occurs, personalizing training loads and recovery protocols based on predictive, data-driven insights (Dudek et al., 2025). This evolution will require a new kind of expertise, blending traditional sports science with data science and machine learning engineering, fundamentally changing the skillset required of practitioners in the field.

### 5.2 The rise of smart textiles and novel sensing modalities

The ultimate vision for a wearable sensor is a device that is entirely imperceptible to the user. The field of smart textiles is bringing this vision closer to reality. Research into weaving conductive yarns, fiber-optic sensors, and even complete computing systems directly into the fabric of clothing represents a paradigm shift in wearable design (Cherenack and Van Pieterson, 2012; Libanori et al., 2022). These sensorized garments promise the ability to conduct continuous, full-body kinematic and physiological monitoring without the encumbrance and attachment issues of the discrete sensor pods used today (Xu et al., 2025). For combat sports, this technology is a potential game-changer, especially for grappling arts like Judo, BJJ, and wrestling, where traditional wearables are impractical due to the intense, full-body contact (Xu et al., 2025). A “smart gi” or “smart rashguard” could provide unprecedented data on the complex biomechanics of throws, pins, and submissions.

Alongside the evolution of form factors, novel sensing modalities are emerging that will provide new layers of data. While IMUs and sEMG are currently dominant, technologies like optical myography (OMG), which uses near-infrared light to measure muscle deformation and activity non-electrically, offer an alternative that may be less susceptible to sweat and electrical artifacts (Nissler et al., 2015; Muhammed and Raghavendra, 2015). Furthermore, the development of wearable biosensors capable of real-time chemical analysis of sweat could provide direct insight into an athlete’s metabolic state, hydration levels, and electrolyte balance (Qiao et al., 2020; Brothers et al., 2019). Fusing this biochemical data with biomechanical and physiological metrics would create a truly comprehensive picture of an athlete’s condition.

### 5.3 Towards personalized biomechanical profiling and injury prevention

The convergence of these advancements—multi-sensor fusion, predictive AI, and seamless smart textiles—points toward a future of highly personalized biomechanical profiling (Dudek et al., 2025). The current approach often relies on comparing an athlete to group averages or idealized models. The future lies in creating a unique “biomechanical passport” for each individual athlete. By continuously monitoring an athlete with integrated wearable systems throughout their training and career, it will be possible to build a longitudinal database of their unique movement signatures, their physiological responses to different training stimuli, and their specific biomechanical risk factors for injury.

This personalized approach will enable training and medical interventions of unprecedented precision. Instead of generic strength and conditioning programs, athletes will receive regimens tailored to address their specific biomechanical inefficiencies or muscular imbalances. Technique coaching will be guided by objective data showing how small adjustments affect an individual’s power output and joint loading. Return-to-play protocols after an injury will be based not on timelines, but on the athlete’s ability to demonstrate that their biomechanical function has returned to their pre-injury baseline. This data-driven, individualized paradigm holds the ultimate promise of maximizing each athlete’s performance potential while simultaneously extending their career longevity.

Despite the promising prospects of personalized biomechanical analysis, the ethical dilemmas it triggers demand urgent and proactive engagement from the sports world. The current ambiguity surrounding data ownership—particularly the urgent need to clarify ownership of the continuous biomechanical data that constitutes an athlete’s “digital twin”—requires resolution; we advocate for an athlete-centric model based on specific, time-limited, and revocable informed consent, safeguarded by data anonymization and encryption to ensure privacy. Predictive analytics could lead to biomechanical discrimination, such as using genetically linked injury-risk data in contract negotiations, while unequal access to technology may foster “technological doping” and undermine competitive fairness, necessitating collective agreements to prohibit data misuse and regulate in-competition real-time feedback. Confronting a regulatory vacuum, we call for a mandatory framework that includes algorithm transparency and auditability, guidelines for head-impact sensor usage (restricted to exposure monitoring, not concussion diagnosis), and independent ethics committees. Importantly, this data-intensive future raises deeper ethical concerns—disputes over the ownership of an athlete’s “biomechanical passport,” *de facto* penalties based on biometric data, and competitive inequities exacerbated by the technological divide—all of which transcend technical issues and require leagues, player associations, and researchers to collaboratively build a robust governance framework. This ensures technology application consistently adheres to fairness and accountability principles: the sustainable development of wearable technology depends not only on innovation but, crucially, on forward-looking governance to establish trust, ensuring performance gains do not come at the cost of athlete wellbeing, privacy, and fair competition.

### 5.4 Concluding synthesis: the transformative potential of wearable sensors in combat sports

The integration of wearable sensor technology has initiated an irreversible shift in combat sports science, moving the field from a reliance on subjective intuition toward a paradigm of objective, data-driven analysis. This review has charted the landscape of this transformation, highlighting the significant progress made in quantifying the biomechanics of striking, where validated systems now provide reliable, in-field measurement of performance metrics such as force, velocity, and technique efficiency. The application of machine learning has further accelerated this progress, enabling the automation of technique classification with high accuracy and paving the way for more efficient performance analysis.

Despite these achievements, formidable challenges persist. The critical hurdles of sensor validation and methodological standardization must be systematically addressed to ensure the scientific rigor and comparability of research across the field. The complex, multi-body dynamics of grappling remain a largely unsolved problem, representing a major frontier for technological and algorithmic innovation. Furthermore, the controversy surrounding head impact sensors underscores the crucial need for responsible science communication to distinguish between their validated use for exposure monitoring and their unproven utility for clinical diagnosis.

Looking forward, the field is poised for another leap, driven by the convergence of multi-sensor fusion, predictive artificial intelligence, and the advent of smart textiles. These technologies promise a future of holistic, seamless, and personalized athlete monitoring. The ultimate goal is to move beyond descriptive analysis and toward a prescriptive and predictive science that can optimize training, prevent injury, and deepen our fundamental understanding of the remarkable biomechanics of human combat. While the path forward is complex and laden with both technical and ethical challenges, the transformative potential of wearable sensor technology to enhance the performance, safety, and longevity of combat athletes is undeniable.

### 5.5 Addressing the grappling gap: sensor design and metric innovation

To address the critical research gap in grappling sports like Judo and BJJ, future efforts must pivot towards purpose-built sensor solutions and novel analytical frameworks. The development of flexible, ruggedized sensors and their integration into smart textiles (e.g., sensor-embedded gis or rash guards) is essential to withstand high-contact environments and prevent dislodgement. Concurrently, research should define and validate new metrics such as “takedown efficiency”—quantifying the relationship between an attacker’s kinetic output and opponent displacement—and “control pressure distribution” across the body during ground engagements. Furthermore, overcoming the complexity of continuous, multi-body interactions requires advanced machine learning approaches, including spatio-temporal models like Graph Neural Networks to analyze dyadic interactions and multi-agent sensing systems to fuse data from both athletes. By focusing on these targeted innovations, the field can transition from acknowledging the gap to actively quantifying the intricate biomechanics of grappling.
